# Factors associated with future dental care utilization among low-income smokers overdue for dental visits

**DOI:** 10.1186/s12903-018-0646-8

**Published:** 2018-11-01

**Authors:** Paula R. Blasi, Chloe Krakauer, Melissa L. Anderson, Jennifer Nelson, Terry Bush, Sheryl L. Catz, Jennifer B. McClure

**Affiliations:** 1 Kaiser Permanente Washington Health Research Institute, 1730 Minor Ave, Suite 1600, Seattle, WA 98101 USA; 2 Department of Biostatistics, University of Washington, F-600, Health Sciences Building 1705 NE Pacific Street, Seattle, WA 98195 USA; 3 Optum Center for Wellbeing Research, 999 3rd Ave., Suite 2000, Seattle, Washington, 98104 USA; 4 Betty Irene Moore School of Nursing, University of California-Davis, 2450 48th Street, Suite 2600, Sacramento, CA 95817 USA

**Keywords:** Oral health, Oral health care, Dental care, Smoking, Tobacco, Dental insurance, Access to dental care, Dental care barriers, Motivation, Self-efficacy

## Abstract

**Background:**

Smokers are at increased risk of oral disease. While routine dental care can help prevent and treat oral health problems, smokers have far lower rates of dental care utilization compared with non-smokers. We sought to better understand which factors may facilitate or hinder dental care utilization among low-income smokers participating in a randomized intervention trial in order to inform future intervention planning.

**Methods:**

This is a secondary analysis of data collected between 2015 and 2017 as part of the OralHealth4Life trial. Participants were eligible callers to the Louisiana, Nebraska, and Oregon state tobacco quitlines who had no dental appointment in the prior or upcoming six months. We examined the association between participants’ baseline characteristics and their receiving professional dental care between baseline and the 6-month follow-up survey.

**Results:**

Participants were racially diverse (42% non-White) and two-thirds had an annual household income under $20,000. Most (86.7%) had not had a dental cleaning in more than one year. Commonly cited barriers to dental care included cost (83.7%) and no dental insurance (78.1%). Those with dental insurance were more likely to see a dentist at follow-up (RR 1.66). Similarly, those reporting a dental insurance barrier to care were less likely to see a dentist at follow-up (RR 0.69); however, there was no significant utilization difference between those reporting a cost barrier vs. those who did not. After controlling for these financial factors, the following baseline characteristics were significantly associated with a higher likelihood of dental care utilization at 6 months: higher motivation (RR 2.16) and self-efficacy (RR 1.80) to visit the dentist, having a disability (RR 1.63), having a higher education level (RR 1.52), and having perceived gum disease (RR 1.49). Factors significantly associated with a lower likelihood of dental care utilization included being married (RR 0.68) and not having a last dental cleaning within the past year (RR 0.47).

**Conclusions:**

Our findings provide important insight into factors that may facilitate or deter use of professional dental care among low-income smokers. This information could inform the development of future interventions to promote dental care utilization.

**Trial registration:**

ClinicalTrials.gov: NCT02347124; registered 27 January 2015.

## Background

Oral health is an important component of overall health and well-being [1]. However, many population groups face a disproportionate burden of oral health problems. Low-income individuals, those with limited education, non-White racial and ethnic groups, and smokers all face higher rates of oral disease than the general population [1–4]. Smokers are a particularly high-risk group because tobacco use is a risk factor for periodontal disease, tooth loss, and oral and pharyngeal cancers [1]. Moreover, smokers are about twice as likely as non-smokers to have not seen a dentist in more than 5 years or to have never seen one [5, 6].

Promoting and facilitating regular dental visits is one strategy to address oral health disparities, as routine dental care can help prevent and treat oral disease [7, 8]. For smokers, routine dental visits may even lessen the negative effects of smoking on oral health because dental providers can evaluate smokers’ oral health, check for early signs of oral and pharyngeal cancers, [6] clean their teeth, and counsel them about oral hygiene behaviors such as brushing and flossing [9]. However, despite the potential benefits of dental visits for smokers [6, 9], there is little research on best practices for improving dental care utilization among this high-risk population.

To address this knowledge gap, we sought to promote dental care utilization among smokers calling state-funded tobacco quitlines. State quitlines offer free phone-based counseling and other assistance to support callers in quitting smoking [10]. Quitline callers include many populations facing oral health disparities, including low-income individuals, those with limited education, and racial or ethnic groups known to have higher rates of oral disease and lower oral cancer survival rates [11, 12]. Quitline callers also have low rates of dental care utilization, [13] and both quitline callers and quitline providers support the idea of integrating oral health promotion content into quitlines [10, 13, 14]. In addition, smokers who call tobacco quitlines presumably are already interested in behavior change, and therefore may be receptive to interventions to promote dental care.

Together with the quitlines and a team of behavioral scientists and oral health experts, we designed an intervention (OralHealth4Life, or OH4L) to encourage dental care utilization among smokers who are overdue for dental visits. The intervention included oral health counseling from quitline coaches, referral information for local low-cost dental providers, and other motivational and educational content. However, the OH4L intervention did not increase dental care utilization compared to usual quitline care in a randomized controlled trial. About 18% of both the intervention and control groups reported seeing a dentist during the 6 months following study enrollment [15]. This finding suggests that smokers, particularly those who are more socio-economically disadvantaged and have multiple barriers to care, may require a different type of intervention to increase their use of professional dental care.

There is little existing research on predictors of future dental visits among low-income smokers or on strategies for increasing dental care utilization among low-income smokers who are overdue for dental care. According to Andersen’s Behavioral Model of Health Services Use, predictors of health care utilization include predisposing factors (such as demographic characteristics and health beliefs), enabling factors (such as financial resources and access to health insurance), and the need for health services [16, 17]. Thus, the goal of the current analysis was to better understand which potential predisposing and enabling factors may facilitate or hinder future dental care utilization among smokers who are overdue for dental care. Based on prior research, we anticipated that lacking dental insurance, self-reporting dental insurance as a barrier, and self-reporting cost as a barrier would be associated with a lower likelihood of seeing a dentist, as previous research shows that a lack of dental insurance and cost are the most commonly cited reasons for forgoing needed dental care [5, 18–20]. Therefore, a primary aim of this analysis was to assess the influence of additional factors on dental care utilization, after accounting for these financial barriers. Based on prior research, we hypothesized that individuals with lower incomes, those with lower education levels, and those living in rural areas would be less likely to see a dentist [6, 21–23]. Based on common health promotion theories and supporting research, we also hypothesized that individuals with higher levels of motivation and self-efficacy for seeing a dentist would be more likely to do so [24–29]. Ultimately, this work seeks to provide important insight into which smokers are more or less likely to see dental care providers, which in turn, could help public health professionals and policymakers design more effective interventions for these individuals in the future.

## Methods

### Overview

This is a secondary analysis of data collected between June 2015 and March 2017 as part of the OH4L trial. Since the main trial did not find a significant treatment effect, we combined intervention groups for this cohort data analysis. Additional trial details can be found in the published protocol, [30] at ClinicalTrials.gov (NCT02347124), and in the results from the main trial [15]. The Kaiser Permanente Washington Institutional Review Board approved this study.

### Participants

Callers to the Oregon (OR), Nebraska (NE), and Louisiana (LA) state tobacco quitlines were invited to be screened for eligibility following registration for services. Callers were eligible to be screened if they were age 18 or older, could read and speak in English, smoked at least 5 cigarettes daily, were ready to quit smoking, and were eligible for their state’s multi-call quitline program. To be eligible for the study, participants also had to have at least some of their natural teeth, no dental appointment in the prior or upcoming 6 months, an interest in improving their oral health, access to the internet, and the ability to receive text messages. We excluded individuals who were incarcerated, were receiving inpatient substance abuse treatment, had significant cognitive impairment or psychosis, were unable to read small text, had plans to move in the next 6 months, or had a household member enrolled in the study.

### Consent, enrollment, and baseline data collection

Eligible callers provided verbal consent, completed the baseline assessment, and were randomized to the control or intervention arm. Randomization was stratified by participants’ dental insurance (yes vs. no/unsure) and quitline (LA, NE, or OR).

### Intervention

Intervention details are available in the published protocol [30]. Briefly, the control group received the standard quitline program, which included 4 to 5 calls with a quitline coach and mailed and online smoking cessation content. Controls also received an attention-matched text messaging intervention focused on generic health promotion tips, excluding smoking cessation or oral health. The experimental group received the standard quitline program plus additional scripted oral health counseling during each quitline call, mailed and online oral health promotion materials, and text messages focused on oral health promotion. The oral health counseling and materials discussed the benefits of oral health care and provided referral information for local low-cost dental providers. To prevent treatment contamination, different quitline counselors treated control and experimental participants. Finally, experimental participants each received a toothbrush, dental floss, and xylitol gum.

### Assessment measures

Demographic data included age, gender, race/ethnicity, state of residence (LA, NE or OR), education, income, disability status, and marital status (single vs. married or living as married). Geographic classification was assessed using the U.S. Department of Agriculture’s rural-urban commuting area (RUCA) codes, [31] based on participant street address. We followed previously used definitions [32] of “urban” as RUCA code 1, “suburban” as RUCA codes 2–6, and “rural” as RUCA codes 7–10.

Oral health-related assessments were self-reported and included standardized items from the 2011–2012 National Health and Nutrition Examination Survey (NHANES), [33] the 2008 National Health Interview Survey (NHIS), [34] and the 2008 Behavioral Risk Factor Surveillance System (BRFSS) [35]. Participants reported whether they thought they had gum disease (yes/no) after being informed that symptoms of gum disease included loose teeth or swollen, receding, sore, or infected gums. Responses of “I don’t know” were coded as not having gum disease. Dental insurance status was assessed by asking participants whether they had dental insurance (yes/no, with responses of “I don’t know” coded as not having dental insurance). Participants also reported length of time since their last dental cleaning (ranging from “in the last 6 months” to “never” on a six-point Likert scale.)

In addition, perceived barriers to dental care were assessed by asking participants to rate the extent to which they believed each of 11 common barriers – such as cost or no dental insurance – affected their ability to seek dental care. Participants rated each barrier on a five-point Likert scale ranging from 1 (“definitely not true”) to 5 (“definitely true”) to indicate the extent to which they perceived that factor as affecting their ability to see a dentist. For our analysis and for ease of interpretation, we grouped similar barriers into the following categories: access (unable to find a dentist, too difficult to get to the clinic), psychological (fear or nervousness, dislike going to the dentist), and prioritization (mean to go but put it off, other health concerns are more important, have no problems with teeth or gums, don’t have time, do not think of it). We reported the cost barrier and no dental insurance barrier separately, as existing literature suggests these barriers affect rates of dental care utilization [5, 18–20]. If a participant rated any individual barrier or barrier in a category as present (rating of 4 or 5), we deemed that individual barrier or category of barrier to be present for that participant. It was possible for participants to report cost or a lack of dental insurance as perceived barriers to dental care, even if they also reported having dental insurance. This situation might arise if a participant had dental insurance that covered few services or involved high premiums and copayments.

Participants rated their motivation and self-efficacy to see a dentist in the next 6 months on a five-point Likert scale ranging from 1 (“not at all motivated/confident”) to 5 (“very motivated/confident”). We deemed ratings of 4 and 5 as “high” motivation or self-efficacy and ratings of 3 or lower as “low” motivation or self-efficacy.

For this analysis, the primary outcome measure was receipt of professional dental care between study enrollment and the 6-month follow-up survey. Receipt of professional dental care was self-reported using an item from the 2012 NHIS, which assessed time since last seeing a dentist, orthodontist, oral surgeon, or dental hygienist [36]. We defined utilization as reporting a dental visit in the prior 6 months at the 2-month or 6-month follow-up survey. Since we excluded individuals from the study who had seen a dentist in the prior 6 months at enrollment, any dental visits reported during the study period represent post-enrollment care. To encourage accurate reporting, we used a modified bogus pipeline methodology [30] which asked participants to provide the contact details for the dental provider they visited. Participants were aware they would be asked for this information if they reported seeing a dental provider during the study period. More than 90% of participants who reported seeing a dentist at 6 months provided contact information for their provider [15].

### Analysis

In the original randomized trial, dental care utilization did not differ significantly by treatment arm at 6-month follow-up, [15] thus we pooled all participants in the current analysis. We used descriptive statistics to characterize the demographic and oral health characteristics of study participants and their baseline barriers to seeing a dental provider.

Our primary analytic goal was to explore the effects of multiple baseline characteristics of interest (e.g., motivation to see a dentist and timing of last dental cleaning) on dental care utilization after accounting for a priori identified financial variables (dental insurance status, dental insurance barrier, cost barrier) and important potential confounders (age, gender, and state) of these associations. As a preliminary step to first understand the influence of each financial barrier previously shown to be associated with dental care utilization (dental insurance status, a dental insurance barrier, and a cost barrier), [5, 18–20] we estimated the relative risk (RR) of dental care receipt within 6 months of follow-up (yes/no) using a separate, unadjusted loglinear regression model with robust standard errors for each financial factor (i.e., using single variable adjusted models). We used this same approach to assess the unadjusted association between receipt of dental care and each potential demographic confounder (age, gender, and state).

Then, to assess the independent effect of each baseline characteristic on future dental care utilization above-and-beyond these financial factors and potential confounders, we used a 2-step process. In step 1, we calculated the RR of seeing a dentist at any time between baseline and the 6-month follow-up for each baseline characteristic of interest using a separate loglinear regression model with robust standard errors. Each model was adjusted for the a priori financial and demographic factors described above (dental insurance status, dental insurance barrier, cost barrier, age, gender, and state). Analyses were limited to participants providing baseline data for each item of interest.

In step 2, we fit one multivariable adjusted loglinear regression model with robust standard errors that included the a priori financial and demographic factors plus all the baseline characteristics found in step 1 to be associated with dental care utilization at the *p* ≤ 0.10 level.

We report 95% confidence intervals for each RR estimate and *p*-values for Wald tests. All confidence intervals and tests use robust standard errors and all analytic results were produced using the *geepack* package in R [37–39].

As a sensitivity analysis, we repeated both step 1 and step 2, limiting the analytic sample to individuals who had not had a dental cleaning in the past year at baseline. The rationale for this analysis was that this subgroup may be more hesitant to seek dental care, and therefore may have different baseline characteristics associated with seeking care.

## Results

### Baseline demographic characteristics

Our final analytic sample consisted of 718 participants from Louisiana (73%), Nebraska (13%) and Oregon (13%) (Table 1). Most participants (59.7%) smoked 21 or more cigarettes per day. Nearly two-thirds had an annual household income under $20,000, and 55.0% had a high school education or less. Participants were racially diverse (58.4% White, 29.1% Black, and 12.5% other or multiple races), and most (87.6%) lived in an urban or suburban area. Nearly all participants were younger than age 65 (mean age 44.3; standard deviation 12.2), and 61.8% were female. Our sample was representative of typical state quitline callers, with the exception that a higher proportion of study participants were non-White [40]. Baseline characteristics by state are presented in Table 1.

**Table 1:** Baseline demographic characteristics of tobacco quitline callers in OH4L study by state of residence, *n* = 718

		Louisiana *N* = 527 N (%)	Nebraska *N* = 95 N (%)	Oregon *N* = 96 N (%)	Overall *N* = 718 N (%)
Age categories	18–44	237 (45.1)	45 (48.4)	53 (55.2)	335 (46.9)
	45–64	271 (51.5)	45 (48.4)	35 (36.5)	351 (49.1)
	65 or older	18 (3.4)	3 (3.2)	8 (8.3)	29 (4.1)
Cigarettes per day	10 or few	51 (9.7)	9 (9.5)	10 (10.5)	70 (9.8)
	11 to 20	149 (28.4)	36 (37.9)	33 (34.7)	218 (30.5)
	21 or more	324 (61.8)	50 (52.6)	52 (54.7)	426 (59.7)
Gender	Male	195 (37.1)	30 (31.6)	49 (51.0)	274 (38.2)
	Female	331 (62.9)	65 (68.4)	47 (49.0)	443 (61.8)
Marital status	Single	303 (58.0)	54 (56.8)	57 (60.0)	414 (58.1)
	Married or living as married	219 (42.0)	41 (43.2)	38 (40.0)	298 (41.9)
Hispanic/Latino	Not Hispanic or Latino	508 (97.3)	93 (97.9)	90 (94.7)	691 (97.1)
	Hispanic or Latino	14 (2.7)	2 (2.1)	5 (5.3)	21 (2.9)
Race/ethnicity	White	260 (49.5)	80 (84.2)	77 (81.9)	417 (58.4)
	Black	201 (38.3)	6 (6.3)	1 (1.1)	208 (29.1)
	Other or multi-racial	64 (12.2)	9 (9.5)	16 (17.0)	89 (12.5)
Geographic classification	Rural	51 (9.7)	27 (28.4)	11 (11.5)	89 (12.4)
	Suburban	140 (26.6)	22 (23.2)	26 (27.1)	188 (26.2)
	Urban	336 (63.8)	46 (48.4)	59 (61.5)	441 (61.4)
Education	GED, HS Degree, or less	307 (59.3)	42 (44.2)	40 (42.6)	389 (55.0)
	At least some college, technical, or trade school	211 (40.7)	53 (55.8)	54 (57.4)	318 (45.0)
Currently employed	Not employed	274 (52.1)	60 (63.2)	51 (53.1)	385 (53.7)
	Employed	252 (47.9)	35 (36.8)	45 (46.9)	332 (46.3)
Living with a disability	No disability	387 (74.0)	57 (60.6)	68 (71.6)	512 (71.9)
	Disability	136 (26.0)	37 (39.4)	27 (28.4)	200 (28.1)
Annual household income	Less than $20,000	310 (61.6)	69 (74.2)	51 (54.3)	430 (62.3)
	More than $20,000	193 (38.4)	24 (25.8)	43 (45.7)	260 (37.7)
Dental insurance status	No dental insurance	390 (74.0)	43 (45.3)	50 (52.1)	483 (67.3)
	Dental insurance	137 (26.0)	52 (54.7)	46 (47.9)	235 (32.7)
Last dental cleaning	Less than 1 year	65 (12.4)	13 (13.8)	16 (16.8)	94 (13.2)
	Over 1 year ago or never	459 (87.6)	81 (86.2)	79 (83.2)	619 (86.8)
Perceived gum disease	No perceived gum disease	322 (61.1)	56 (58.9)	50 (52.1)	428 (59.6)
	Perceived gum disease	205 (38.9)	39 (41.1)	46 (47.9)	290 (40.4)
Motivation to see dentist in next 6 months	Low motivation	88 (16.7)	21 (22.1)	25 (26.0)	134 (18.7)
	High motivation	439 (83.3)	74 (77.9)	71 (74.0)	584 (81.3)
Self-efficacy to see dentist in next 6 months	Low self-efficacy	168 (31.9)	30 (31.6)	39 (40.6)	237 (33.0)
	High self-efficacy	359 (68.1)	65 (68.4)	57 (59.4)	481 (67.0)
Dental insurance barrier	No such barrier	88 (16.8%)	42 (44.2%)	27 (28.1%)	157 (21.9%)
	Dental insurance barrier	437 (83.2%)	53 (55.8%)	69 (71.9%)	559 (78.1%)
Cost barrier	No such barrier	74 (14.1%)	23 (24.5%)	20 (20.8%)	117 (16.3%)
	Cost barrier	452 (85.9%)	71 (75.5%)	76 (79.2%)	599 (83.7%)
Psychological barriers^a^	No such barriers	247 (46.9)	35 (36.8)	46 (47.9)	328 (45.7)
	Psychological barriers	280 (53.1)	60 (63.2)	50 (52.1)	390 (54.3)
Prioritization barriers^a^	No such barriers	128 (24.3%)	19 (20.0%)	29 (30.2%)	176 (24.5%)
	Prioritization barriers	399 (75.7%)	76 (80.0%)	67 (69.8%)	542 (75.5%)
Access barriers^a^	No such barriers	349 (66.2%)	66 (69.5%)	66 (68.8%)	481 (67.0%)
	Access barriers	178 (33.8%)	29 (30.5%)	30 (31.2%)	237 (33.0%)

Missing values: Age (3); Cigarettes per day (4); Gender (1); Marital status (6); Hispanic/Latino (6); Race/ethnicity (4); Education (11); Currently employed (1); Living with a disability (6); Annual household income (28); Last dental cleaning (5)

^a^For our analysis and for ease of interpretation, we grouped similar barriers into the following categories: access (unable to find a dentist, too difficult to get to the clinic), psychological (fear or nervousness, dislike going to the dentist), and prioritization (mean to go but put it off, other health concerns are more important, have no problems with teeth or gums, don’t have time, do not think of it)

### Baseline oral health-related characteristics and self-reported barriers

At baseline, most participants (86.8%) had not had a dental cleaning in more than 1 year (Table 1) and 41.7% had not had one in more than 5 years or had never had one (data not shown). Most participants reported high levels of motivation (81.3%) and self-efficacy (67.0%) for visiting a dentist in the next six months. Only about one-third of participants reported having dental insurance, 83.7% cited cost as a barrier to obtaining dental care, and 78.1% cited a dental insurance barrier. Many participants (75.5%) reported one or more prioritization barriers, such as meaning to go but putting it off, or viewing other health concerns as more important. About half of participants (54.3%) reported psychological barriers, which included fear or nervousness and disliking dental visits. Only about one-third (33.0%) of participants reported access-related barriers, such as an inability to find a dentist or difficulty getting to the office or clinic.

### Association of financial barriers and potential demographic confounders with dental care utilization

When we examined each of our a priori identified financial and demographic factors (dental insurance status, dental insurance barrier, cost barrier, age, gender, and state), we found no association between dental care utilization at 6-month follow-up and either age, gender, or state (Table 2**)**. Consistent with prior research, dental insurance status and perceived dental insurance barriers were associated with receipt of dental care. Participants with dental insurance were more likely to have seen a dentist by 6-month follow-up than those without (RR 1.66 [95% CI 1.22–2.26]). Participants who reported having a dental insurance barrier to care were less likely to have seen a dentist compared with those who did not report this barrier (RR 0.69 [95% CI 0.50–0.97]). Of those who reported cost as a barrier to dental care, 17.5% had seen a dentist compared with 23.1% of those who did not report this barrier; however, the cost barrier was not significantly associated with dental care utilization (RR 0.76 [95% CI 0.52–1.10]).

**Table 2:** Association of a priori identified financial and demographic confounders with dental utilization by 6-month follow-up

		Use of Dental Care, %	Unadjusted RR (95% CI)	*p*-value
Age	18 to 44	16.7	1 (ref)	0.661
	45 to 64	19.4	1.16 (0.84–1.60)	
	65 or older	17.2	1.03 (0.45–2.37)	
Gender	Male	17.2	1 (ref)	0.496
	Female	19.2	1.12 (0.81–1.55)	
State	Louisiana	16.5	1 (ref)	0.090
	Nebraska	24.2	1.47 (0.98–2.20)	
	Oregon	22.9	1.39 (0.92–2.10)	
Dental insurance status	No dental insurance	15.1	1 (ref)	**0.001**
	Dental insurance	25.1	1.66 (1.22–2.26)	
Dental insurance barrier	No such barrier	24.2	1 (ref)	**0.030**
	Dental insurance barrier	16.8	0.69 (0.50–0.97)	
Cost barrier	No such barrier	23.1	1 (ref)	0.144
	Cost barrier	17.5	0.76 (0.52–1.10)	

Note Bolded text indicates associations that are significant at the *p* < 0.05 level

### Associations between baseline characteristics of interest and dental care utilization

In the single variable adjusted models (Table 3), we found that factors significantly associated with a higher likelihood of dental care utilization at followup included having high levels of motivation to see the dentist (RR 2.86 [95% CI 1.55–5.29]) and high self-efficacy for seeing the dentist (RR 2.26 [95% CI 1.46–3.49]). Participants with a disability were more likely to see a dentist than those without a disability (RR 1.65 [95% CI 1.17–2.32]), and those who had at least some college education were more likely to see a dentist compared with those who had a high school education or less (RR 1.63 [95% CI 1.17–2.26]). Factors significantly associated with a lower likelihood of dental care utilization at followup including being married (RR 0.68 [95% CI 0.48–0.95]), and not having received a dental cleaning in more than 1 year (RR 0.47 [95% CI 0.33–0.67]).

**Table 3:** Associations between baseline characteristics and dental utilization by 6-month follow-up in single variable adjusted models^a^

		Use of Dental Care, %	Adjusted RR (95% CI)^a^	*p*-value
Marital status	Single	21.0	1 (ref)	**0.023**
	Married or living as married	14.4	0.68 (0.48–0.95)	
Education	GED, HS Degree, or less	14.4	1 (ref)	**0.004**
	At least some college, technical, or trade school	23.9	1.63 (1.17–2.26)	
Living with a disability (employed & unemployed)	No disability	15.2	1 (ref)	**0.005**
	Disability	26.5	1.65 (1.17–2.32)	
Last dental cleaning	Less than 1 year	36.2	1 (ref)	**< 0.001**
	Over 1 year ago or never	15.7	0.47 (0.33–0.67)	
Perceived gum disease	No perceived gum disease	15.8	1 (ref)	0.066
	Perceived gum disease	22.1	1.35 (0.98–1.85)	
Motivation to see dentist in next 6 months	Low motivation	7.5	1 (ref)	**0.001**
	High motivation	20.9	2.86 (1.55–5.29)	
Self-efficacy to see dentist in next 6 months	Low self-efficacy	9.7	1 (ref)	**< 0.001**
	High self-efficacy	22.7	2.26 (1.46–3.49)	
Cigarettes per day	10 or fewer	20	1 (ref)	0.95
	11 to 20	19.3	0.92 (0.54–1.58)	
	21 or more	17.8	0.93 (0.56–1.55)	
Hispanic/Latino	Not Hispanic or Latino	18.7	1 (ref)	0.35
	Hispanic or Latino	9.5	0.52 (0.13–2.07)	
Race/ethnicity	White	20.1	1 (ref)	0.21
	Black	16.8	0.92 (0.62–1.36)	
	Other or multi-racial	10.1	0.56 (0.29–1.07)	
Geographic classification	Rural	21.3	1 (ref)	0.73
	Suburban	19.7	0.10 (0.61–1.6)	
	Urban	17.2	0.88 (0.56–1.39)	
Currently employed	Not employed	19	1 (ref)	0.72
	Employed	17.5	0.94 (0.68–1.30)	
Annual household income	Less than 20 K	19.5	1 (ref)	0.25
	Over 20 K	16.5	0.82 (0.58–1.15)	
Psychological barriers	No such barriers	19.2	1 (ref)	0.31
	Psychological barriers	17.7	0.85 (0.61–1.17)	
Prioritization barriers	No such barriers	15.9	1 (ref)	0.61
	Prioritization barriers	19.2	1.11 (0.75–1.63)	
Access barriers	No such barriers	18.3	1 (ref)	0.99
	Access barriers	18.6	1.00 (0.72–1.38)	

Bolded text indicates associations that are significant at the *p* < 0.05 level

^a^A separate model assesses each baseline characteristic of interest one at a time. All models are adjusted for a priori identified financial variables (dental insurance status, dental insurance barrier, cost barrier) and potential confounders (age, gender, and state). All *p*-values are global *p*-values

All associations that were significant in our single variable models (Table 3) remained significant in our multivariable model (Table 4), though RR estimates were somewhat attenuated (RRs 2.16 for motivation, 1.80 for self-efficacy, 0.52 for having a recent dental cleaning, 1.52 for having a higher education level, 0.67 for being married, and 1.63 for living with a disability). The association between perceived gum disease and dental care utilization was near-significant in our single variable models (RR 1.35 [95% CI 0.98–1.85]) and became significant with multivariable adjustment (RR 1.49 [95% CI, 1.09–2.05]).

**Table 4:** Associations between baseline characteristics and dental utilization by 6-month follow-up in multivariable adjusted model^a^

		Use of Dental Care, %	Adjusted RR (95% CI)^a^	*p*-value
Age	18 to 44	16.7	1 (ref)	0.96
	45 to 64	19.4	0.97 (0.69–1.38)	
	65 or older	17.2	0.89 (0.39–2.03)	
Gender	Male	17.2	1 (ref)	0.83
	Female	19.2	0.97 (0.70–1.33)	
State	Louisiana	16.5	1 (ref)	0.45
	Nebraska	24.2	1.14 (0.75–1.75)	
	Oregon	22.9	1.30 (0.86–1.96)	
Dental insurance status	No dental insurance	15.1	1 (ref)	0.33
	Dental insurance	25.1	1.20 (0.84–1.73)	
Dental insurance barrier	No such barrier	24.2	1 (ref)	0.61
	Dental insurance barrier	16.8	0.90 (0.60–1.35)	
Cost barrier	No such barrier	23.1	1 (ref)	0.88
	Cost barrier	17.5	0.97 (0.64–1.46)	
Marital status	Single	21.0	1 (ref)	**0.02**
	Married or living as married	14.4	0.67 (0.48–0.94)	
Education	GED, HS Degree, or less	14.4	1 (ref)	**0.013**
	At least some college, technical, or trade school	23.9	1.52 (1.09–2.12)	
Living with a disability (employed & unemployed)	No disability	15.2	1 (ref)	**0.004**
	Disability	26.5	1.63 (1.17–2.27)	
Last dental cleaning	Less than 1 year	36.2	1 (ref)	**< 0.001**
	Over 1 year ago or never	15.7	0.52 (0.37–0.72)	
Perceived gum disease	No perceived gum disease	15.8	1 (ref)	**0.014**
	Perceived gum disease	22.1	1.49 (1.09–2.05)	
Motivation to see dentist in next 6 months	Low motivation	7.5	1 (ref)	**0.029**
	High motivation	20.9	2.16 (1.08–4.32)	
Self-efficacy to see dentist in next 6 months	Low self-efficacy	9.7	1 (ref)	**0.019**
	High self-efficacy	22.7	1.80 (1.10–2.93)	

Bolded text indicates associations that are significant at the *p* < 0.05 level

^a^Includes our a priori identified covariates (cost barrier, dental insurance barrier, having dental insurance [yes/no], gender, age, and state), as well as each variable found to be significant at the *p* ≤ 0.10 level in the single variable models (step 1). All p-values are global p-values

Age, gender, and state all remained unassociated with dental care utilization in the multivariable model. Contrary to our expectations, we found little to no association in the multivariable model between dental care utilization and any financial factors, including dental insurance status (RR 1.20 [95% CI, 0.84–1.73]), self-reported dental insurance barriers (RR 0.90 [95% CI, 0.60–1.35]), or self-reported cost barriers (RR 0.97 [95% CI, 0.64–1.46]).

### Post-hoc sensitivity and exploratory analyses

In sensitivity analyses conducted among individuals whose last dental cleaning was more than 1 year ago (*n* = 459), the single variable adjusted model results mirrored the full cohort analysis, with no change in the significance of any variables nor the direction of association for any significant variables (data not shown). In the multivariable adjusted model, the estimated associations were also generally in the same direction and of similar magnitude as found in the broader sample, but due to reduced power, many associations were no longer significant in this subgroup (data not shown).

We also conducted exploratory analyses to further examine our unexpected findings that being single and having a disability were associated with receipt of dental care at follow-up. For this analysis, we compared other baseline characteristics between single vs. married participants as well as between participants with a disability vs. those without. We found that a higher proportion of single participants had completed at least some college (48.3% compared to 39.9% among married participants). We also found that a much higher percentage of individuals living with a disability were unemployed (92.0% vs. 38.5% for those without a disability), and that Nebraska had a higher proportion of participants with a disability (39.4%) compared with Oregon (28.4%) and Louisiana (26.0%). However, as shown in the multivariable findings, adjusting for education status and state did not change the significance of the association between either disability status or marital status and dental care utilization.

## Discussion

Smokers, particularly those who are low-income or uninsured, are an important target group for promoting dental care utilization. To better inform future efforts to promote dental visits in this high-risk group, we examined baseline factors associated with future dental care among participants in the OH4L trial. We found that significant predictors of future dental care included being single, not reporting dental insurance as a barrier to care, and having dental insurance, high levels of motivation and self-efficacy to visit the dentist, a disability, a higher education level, perceived gum disease, and a dental cleaning within the past year.

Our finding that having dental insurance was associated with a greater likelihood of seeking future dental care was consistent with research from other countries, including studies from Korea [41] and Finland [42] showing an increase in dental visit attendance after a nationwide expansion in public dental insurance coverage. In the U.S., only about 7% of adults have public dental insurance (provided through Medicaid) and about 60% have private dental insurance, usually obtained through their employer or on the individual insurance market [43]. About 33% of U.S. residents – and about 67% of our study sample – have no dental insurance and therefore bear the cost burden of dental care [43]. In our analysis, we found lower rates of dental care utilization among study participants who reported cost as a barrier to dental care, although this association was not significant. Together, these findings about cost and dental insurance support the notion that financial factors may represent important barriers to accessing dental care, a finding that is consistent with prior research [5, 18–20].

However, we found these financial barriers were no longer significantly associated with the likelihood of seeking dental services after adjusting for other baseline variables. This unexpected finding contrasts with prior research, and suggests financial barriers do little to alter the likelihood of seeking professional dental care after adjusting for other personal characteristics, such as having high levels of motivation and self-efficacy to visit the dentist, perceived gum disease, a more recent dental cleaning, a higher education level, being single, and having a disability.

There are several possible explanations for the discrepancies between prior research and our findings regarding the role of financial factors as barriers to dental care. Most prior studies were conducted among nationally or regionally representative samples of civilian non-institutionalized populations, [18–20] which in some cases were stratified by smoking status [5]. In contrast, our sample consisted exclusively of smokers from Louisiana, Nebraska, and Oregon, most of whom were very low-income. Therefore, the factors that influence dental care utilization among these smokers in our three states might differ from those that influence utilization among a population with a greater diversity of income levels. In addition, most prior studies were cross-sectional analyses that provided the frequencies of participants’ self-reported reasons for forgoing needed dental care without adjusting for other personal characteristics, [5, 19, 20] or assessed the likelihood of different outcomes, such as lack of care for known dental problems [18]. In contrast, our prospective, longitudinal analysis offers insight into the characteristics associated with an increased likelihood of transitioning from being a non-utilizer to a utilizer of dental services, which may involve different influences.

We undertook this analysis to explore factors that might be hindering or facilitating dental visits among high-risk smokers to inform future intervention development. In contrast to our expectations, neither income level nor geographic classification (urban, suburban, rural) were significantly associated with future dental care utilization; however, the former may reflect the lack of economic diversity in our sample. Two-thirds had annual household incomes below $20,000, and less than 5% had annual household incomes over $60,000 [15]. Other factors not associated with future dental care utilization included psychological barriers such as fear or nervousness, access-related barriers such as difficulty finding a dentist, and prioritization barriers such as viewing other health concerns as more important. We initially expected these factors to play a role in dental utilization, and in fact, the OH4L intervention sought to address these issues through a combination of cognitive behavioral counseling, oral health education, and referrals to local low-cost dental providers. However, our comprehensive, multi-modal intervention had no effect on future dental care [15]. Taken together, the results of our randomized trial and the findings from this secondary analysis suggest income, geographic classification, and perceived barriers may be less important drivers of future dental care among low-income smokers than one’s motivation to see a dentist or confidence in one’s ability to see the dentist.

Since higher levels of motivation and self-efficacy to visit the dentist were associated with a greater likelihood of future dental care utilization, we recommend future interventions specifically seek to build individuals’ motivation and self-efficacy to visit the dentist, independent of their concerns about cost. In addition, our finding that people with perceived gum disease were more likely to see a dental provider suggests future interventions should educate smokers about their gum health and oral disease risk while simultaneously fostering their self-efficacy and motivation to see a dental provider. Based on evidence that gain-framed health risk messaging is more persuasive than loss-framed messaging for oral hygiene behaviors, [44–46] we recommend that such an intervention emphasize the positive benefits of seeing a dentist, as opposed to the health consequences of neglecting this care.

Other significant correlates of future dental care included having a higher education level, being single, and having a disability. While the association between dental care utilization and education aligns with prior research, [22] our findings on marital status and disability status were unexpected. Our exploratory analysis found that single participants generally had higher levels of education than married participants; however, adjusting for education level did not impact the significance of the association between marital status and seeking dental care. In addition, participants with a disability were more likely to report being unemployed than participants without a disability, so it is possible that participants with a disability had more free time to visit the dentist. Nebraska also had a higher proportion of participants with a disability compared with Oregon and Louisiana. However, neither employment status nor state were significantly associated with future dental care use in any of our analyses, suggesting the association between disability status and dental care utilization may be complex and not easily explained by our current analyses or available data.

Overall, our findings highlight the difficulty of increasing dental care utilization among high-risk, low-income smokers and the need for additional research to improve our understanding of how to best support tobacco users in obtaining dental care. For example, future studies could interview smokers about what they would need to schedule, attend, and maintain routine dental visits. Such interviews might uncover factors that were not explored in our present analysis but could play a role in supporting dental visit attendance, such as assistance with appointment scheduling, appointment reminders, or childcare during visits.

### Strengths and limitations

This study has several strengths, including its longitudinal, population-based design that allowed us to follow individuals who transitioned from being non-utilizers to utilizers of dental care. It includes a diverse population of smokers interested in quitting (42% non-White) and a high proportion of very low-income smokers. These are priority populations for intervention because of their high risk of oral disease and low rates of dental care utilization, however, they also face substantial barriers to obtaining dental care. Therefore, understanding the characteristics of participants who transitioned from non-utilizers to utilizers of dental care offers valuable insight for future efforts to facilitate dental care utilization among similar high-risk groups.

This study also has certain limitations. Our sample consisted of a low-income population of smokers from Louisiana, Nebraska, and Oregon who were overdue for dental visits, and our findings may not be generalizable to higher-income smokers, those from other regions or countries, those who visit a dentist regularly, or smokers who have not contacted a quitline for help with tobacco cessation. As a condition of enrollment, participants had to be interested in improving their oral health. As such, the results may not generalize to persons with no interest in improving their oral health. All participant data was based on self-report, and thus subject to bias and misreporting. However, for our main outcome of dental care utilization, our methodology was designed to deter misreporting by requiring a provider name. Since more than 90% of participants provided this information with an understanding that we may also ask their permission to contact their provider to verify the accuracy of their report, we have great confidence in the veracity of this self-reported data.

## Conclusions

Low-income smokers are at high risk for oral disease and are a priority group for oral health intervention. The findings from our randomized trial underscore the difficulty of increasing dental care utilization among this population, but the current analysis provides useful insight into those who are more or less likely to seek dental care. This information can inform the design of future oral health promotion programs either by helping public health officials target those at greatest risk for not seeking dental care (e.g., smokers with lower education levels) or by suggesting potential targets for future behavioral interventions (e.g., perceived disease risk, self-efficacy, motivation). Future research should evaluate whether interventions that target these individuals and address these factors can increase dental care utilization among low-income individuals, particularly smokers.

## Abbreviations

BRFSS: Behavioral Risk Factor Surveillance System
LA: Louisiana
NE: Nebraska
NHANES: National Health and Nutrition Examination Survey
NHIS: National Health Interview Survey
OH4L: OralHealth4Life
OR: Oregon
RR: Relative risk
RUCA: Rural-Urban Commuting Area
